# Aspirin as secondary prevention in colorectal cancer liver metastasis (ASAC trial): study protocol for a multicentre randomized placebo-controlled trial

**DOI:** 10.1186/s13063-021-05587-w

**Published:** 2021-09-20

**Authors:** Sheraz Yaqub, Bjørn Atle Bjørnbeth, Jon-Helge Angelsen, Claus Wilki Fristrup, Jon Erik Grønbech, Oskar Hemmingsson, Bengt Isaksson, Ingebjørg Soterud Juel, Peter Nørgaard Larsen, Gert Lindell, Frank Viborg Mortensen, Kim Erlend Mortensen, Magnus Rizell, Per Sandström, Oddvar Mathias Sandvik, Ernesto Sparrelid, Helena Taflin, Kjetil Taskén, The ASAC study group, Kristoffer W. Brudvik, Åsmund A. Fretland, Arild Horn, Dyre Kleive, Knut J. Labori, Kristoffer Lassen, Bård I. Røsok, Jon A. Søreide, Tore Tholfsen, Olaug Villanger, Anne Waage

**Affiliations:** 1 Department of Hepato-Pancreato-Biliary Surgery, Oslo University Hospital, Oslo, Norway; 2 Institute of Clinical Medicine, University of Oslo, Oslo, Norway; 3 Department of Acute and Digestive Surgery, Haukeland University Hospital, Bergen, Norway; 4 Departments of Clinical Medicine, University of Bergen, Bergen, Norway; 5 Department of Surgery, Odense University Hospital, Odense C, Denmark; 6 Department of Gastrointestinal Surgery, St. Olav’s Hospital, Trondheim University Hospital, Trondheim, Norway; 7 Department of Clinical and Molecular Medicine, NTNU, Norwegian University of Science and Technology, Trondheim, Norway; 8 Department of Surgical and Perioperative Sciences, Umeå University, Umea, Sweden; 9 Department of Surgical Sciences, Uppsala University, Uppsala, Sweden; 10 Department of Gastrointestinal Surgery, Rigshospitalet, Copenhagen, Denmark; 11 Department of Surgery, Skåne University Hospital, Lund, Sweden; 12 Department of Surgery, Aarhus University Hospital, Aarhus N, Denmark; 13 Department of Gastrointestinal Surgery, University Hospital of North, Tromsø, Norway; 14 Department of Transplantation, Institute of Clinical Sciences, University of Gothenburg, Sahlgrenska University Hospital, Gothenburg, Sweden; 15 Department of Surgery, County Council of Östergötland, Linköping, Sweden; 16 Department of Biomedical and Clinical Sciences, Division of Surgery, Orthopedics and Oncology, Linköping, Sweden; 17 Department of Gastrointestinal Surgery, Stavanger University Hospital, Stavanger, Norway; 18 Division of Surgery, Department of Clinical Science, Intervention, and Technology (CLINTEC), Center for Digestive Diseases, Karolinska University Hospital, Karolinska Institute, Stockholm, Sweden; 19 Department of Surgery, Institute of Clinical Sciences, University of Gothenburg, Sahlgrenska University Hospital, Gothenburg, Sweden; 20 Institute for Cancer Research, Oslo University Hospital, and Institute of Clinical Medicine, University of Oslo, Oslo, Norway

**Keywords:** Colorectal cancer, Liver metastases, Aspirin, Acetylsalicylic acid, Secondary prevention

## Abstract

**Background:**

Colorectal cancer is one the most common cancers in the western world with increasing incidence. Approximately 50% of the patients develop liver metastases. Resection of liver metastases is the treatment of choice although almost half of the resected patients get recurrence in the liver.

**Methods:**

The ASAC trial is a Scandinavian, multicentre, double-blinded, randomized, placebo-controlled study to determine whether adjuvant treatment with low-dose aspirin (acetylsalicylic acid (ASA)) can improve disease-free survival in patients treated for colorectal cancer liver metastases (CRCLM). Up to 800 patients operated for CRCLM will be randomized to Arm#1 ASA 160 mg once daily or Arm#2 Placebo, for a period of 3 years or until disease recurrence. The patients will be recruited at all major hepatobiliary surgical units in Norway, Sweden and Denmark and have follow-up according to standard of care and the National Guidelines.

**Discussion:**

The ASAC trial will be the first clinical interventional trial to assess the potential beneficial role of ASA in recurrence of CRCLM and survival. ASA is an inexpensive, well-tolerated and easily accessible drug that will be highly potential as adjuvant drug in secondary prevention of CRCLM if the study shows a beneficial effect. We will also determine the effect of ASA as adjuvant treatment on Health-Related Quality of Life and the cost-effectiveness.

**Trial registration:**

ClinicalTrials.gov NCT03326791. Registered on 31 October 2017.

## Administrative information

The order of the items has been modified to group similar items (see http://www.equator-network.org/reporting-guidelines/spirit-2013-statement-defining-standard-protocol-items-for-clinical-trials/).

**Table:** 

Title {1}	Aspirin as secondary prevention in colorectal cancer liver metastasis (ASAC trial): Study protocol for a multicentre randomized placebo-controlled trial
Trial registration {2a and 2b}.	ClinicalTrials.gov Identifier: NCT03326791 Aspirin in Colorectal Cancer Liver Metastases (ASAC)
Protocol version {3}	Protocol version 5.3; March 30, 2020
Funding {4}	Oslo University Hospital, Oslo, Norway Norwegian Cancer Society The Research Council of Norway The national program for clinical therapy research - Norway (KLINBEFORSK)
Author details {5a}	SPIRIT guidance: Affiliations of protocol contributors. Sheraz Yaqub^1,2^, Bjørn Atle Bjørnbeth^1^, Jon-Helge Angelsen^3,4^, Claus Wilki Fristrup^5^, Jon Erik Grønbech^6,7^, Oskar Hemmingsson^8^, Bengt Isaksson^9^, Ingebjørg Soterud Juel^6^, Peter Nørgaard Larsen^10^, Gert Lindell^11^, Frank Viborg Mortensen^12^, Kim Erlend Mortensen^13^, Magnus Rizell^14^, Per Sandström^15,16^, Oddvar Mathias Sandvik^17^, Ernesto Sparrelid^18^, Helena Taflin^19^, Kjetil Tasken^20^, The ASAC study group^#^ ^1^Dept of Hepato-Pancreato-Biliary Surgery, Oslo University Hospital, Oslo, Norway ^2^Institute of Clinical Medicine, University of Oslo, Oslo, Norway ^3^Department of Acute and Digestive Surgery, Haukeland University Hospital, Bergen, Norway. ^4^Departments of Clinical Medicine, University of Bergen, Bergen, Norway. ^5^Department of Surgery, Odense University Hospital, Odense C, Denmark. ^6^Department of Gastrointestinal Surgery, St. Olav's Hospital, Trondheim University Hospital, Trondheim, Norway. ^7^Department of Clinical and Molecular Medicine, NTNU, Norwegian University of Science and Technology, Trondheim, Norway. ^8^Department of Surgical and Perioperative Sciences, Umeå University, Umea, Sweden. ^9^Department of Surgical Sciences, Uppsala University, Uppsala, Sweden. ^10^Department of Gastrointestinal Surgery, Rigshospitalet, Copenhagen, Denmark. ^11^Department of Surgery, Skåne University Hospital, Lund, Sweden. ^12^Department of Surgery, Aarhus University Hospital, Aarhus N, Denmark. ^13^Department of Gastrointestinal Surgery, University Hospital of North, Tromsø, Norway. ^14^Department of Transplantation, Institute of Clinical Sciences, University of Gothenburg, Sahlgrenska University Hospital, Gothenburg, Sweden. ^15^Department of Surgery, County Council of Östergötland, Linköping, Sweden. ^16^Department of Biomedical and Clinical Sciences, Division of Surgery, Orthopedics and Oncology, Linköping, Sweden. ^17^Department of Gastrointestinal Surgery, Stavanger University Hospital, Stavanger, Norway. ^18^Division of Surgery, Department of Clinical Science, Intervention, and Technology (CLINTEC), Center for Digestive Diseases, Karolinska University Hospital, Karolinska Institute, Stockholm, Sweden. ^19^Department of Surgery, Institute of Clinical Sciences, University of Gothenburg, Sahlgrenska University Hospital, Gothenburg, Sweden. ^20^Institute for Cancer Research, Oslo University Hospital, and Institute of Clinical Medicine, University of Oslo, Oslo, Norway.
Name and contact information for the trial sponsor {5b}	SPIRIT guidance: Name and contact information for the trial sponsor. Oslo University Hospital (OUH) Elin Henriksen P.O.Box 4950 Nydalen, 0424 Oslo Tel: + 47-915 02770 E-mail: ehenri@ous-hf.no
Role of sponsor {5c}	SPIRIT guidance: Role of study sponsor and funders, if any, in study design; collection, management, analysis, and interpretation of data; writing of the report; and the decision to submit the report for publication, including whether they will have ultimate authority over any of these activities. The study sponsor and funders have no role or authorities in study design, data collection, management, analysis, or publication.

## Introduction

### Background and rationale {6a}

Colorectal cancer (CRC) is the third most common cancer worldwide, and metastatic disease is the most common cause of death in these patients [1]. Surgical resection is the only potential curative treatment for CRC liver metastases (CRCLM) [2–4]. A number of reports have shown that intake of non-steroidal anti-inflammatory drugs (NSAIDs) or acetylsalicylic acid (ASA) inhibiting cyclooxygenases (COX) reduce the risk of CRC development (primary prevention) [5–7]. Other studies have shown that selective COX-2 inhibitors also are associated with a decline in the incidence of CRC and reduced mortality rate, but adverse cardiovascular events precluded further studies, in particular with rofecoxib [8]. ASA, on the other hand, has a favourable cardiovascular profile and better safety. Recent meta-analyses of 5 to 51 different interventional studies with ASA originally conducted for cardiovascular indications and with 5 to 20 years follow-up have indicated, when they were re-analysed and linked with cancer registry data, that ASA indeed reduced the risk of later CRC (and other gastrointestinal cancers) with hazard ratios of 0.45 to 0.75 for cumulative incidence and mortality and with doses as low as 75 mg per day, and with increasing benefit with duration of treatment [9–11]. Preventive effects of ASA were also seen with shorter duration of ASA treatment, but then only with higher dose than 75 mg per day [10]. In contrast, a large study in the USA (Physicians Health Study, with 22,000 subjects) where ASA was administered every second day did not show effect on CRC development [12]. However, a comparison between meta-analysis of randomized studies, case-control studies, and observational registry studies with daily administration of ASA all showed a similar and highly significant reduction in the risk of CRC (odds ratio 0.54–0.69) for all, indicating a distinct and robust effect of ASA in primary prevention of CRC [13, 14]. These and other studies have led to an on-going discussion about the use of ASA as a cost-effective primary prevention of CRC with added benefit of the cardiovascular effects and versus its safety profile [15–17]. The phosphatidylinositol 3-kinase (PI3K) pathway is frequently altered in CRC, e.g. through mutations of PIK3CA (phosphatidylinositol-4,5-bisphosphonate 3-kinase, catalytic subunit alpha polypeptide gene). Recently, acquired mutations in the PIK3CA gene were shown to predict benefit from treatment with ASA [18, 19]. While patients whose tumours did not carry a PIK3CA mutation had no observed benefit from aspirin therapy in these trials, patients whose tumours carry PIK3CA mutations had a HR of 0.11–0.18 for colorectal cancer-specific death. These data are based on retrospective analyses and require confirmation in prospective randomized trials to establish treatment recommendations for ASA in patients with colorectal cancer.

In aggregate, our studies and others on the mechanisms of action of prostaglandins in CRC and effects of perturbing COX by NSAIDs or ASA [20, 21] argue that further studies on the effect of COX inhibition post-diagnosis in CRC are warranted.

COX-2 levels are elevated in as many as 85% of human CRCs and approximately 50% of colorectal adenomas [22]. Prostaglandin E2 (PGE2) has been shown to be an important mediator of COX-2-associated effects and PGE2 levels are elevated in CRC biopsies compared with normal mucosa. Homozygous deletion of the gene for the PGE2 receptor EP2 that signals through cyclic AMP (cAMP) reduced the number and size of colorectal polyps in a polyposis mouse model [20]. Beside a well-documented pro-angiogenic effect [23], PGE2 promotes apoptosis and stimulates growth of tumour stem cells both through the EP2 and EP3 receptors by synergizing with the Wnt/β-catenin pathway, relevant in cancers where the adenomatous polyposis coli (APC) gene is activated [24]. This leads to translocation of beta-catenin, which turns on a programme of genes including c-myc and COX-2. Furthermore, COX-2 overexpression correlates with tumour recurrence and metastasis of CRC and in this context tumour immunology may be particularly important. Our novel observations in two clinical observational studies show that the PGE2 produced also inhibits anti-tumour immunity [25, 26]. Hence, the effect of COX inhibition to perturb PGE2 signalling in established CRC would be 3-fold: inhibiting angiogenesis and tumour growth and stimulating anti-tumour immunity.

In terms of anti-tumour immunity, a growing tumour with several activating mutations should normally be recognized as foreign by the immune system and eliminated. However, by cancer immunoediting, tumour cells with additional mutations that activate various evasion mechanisms are selected [27]. These mechanisms encompass (i) downregulation of MHC-I to avoid recognition; (ii) overexpression of Indoleamine 2,3 dioxygenase (IDO) to induce tolerance; (iii) activation of inhibitory co-receptors on immune cells or triggers of apoptosis; (iv) recruitment and expansion of regulatory T cells (Tregs); and (v) secretion of immunosuppressive tumour-derived soluble factors such as PGE2 that inhibit immune responses [28]. Interestingly, several tumour evasion mechanisms such as secretion of PGE2 from tumour cells and several suppressive mechanisms by Tregs converge on cAMP immune suppression in effector T cells [20].

Our data show that continuous activation of T cells leads to generation of adaptive Tregs [29]. Adaptive iTregs express COX-2 as a consequence of continuous exposure to antigen, leading to secretion of PGE2. PGE2 in turn stimulates FOXP3 expression in the Tregs and inhibits effector T cell function through activation of the cAMP inhibitory pathway [30]. Finally, LPS-activated monocytes also secrete high levels of PGE2, inhibiting T cell activation and inducing FOXP3 expression [31].

Cyclic AMP and protein kinase A (PKA) are involved in the regulation of a broad range of body functions and most organ systems. Cyclic AMP acts as an acute inhibitor of T cell activation that prevents T cell proliferation and cytokine production. We have previously demonstrated that T effector immune suppression can be reversed by small molecule pharmaceutical inhibitors of the cAMP signalling pathway in normal blood donors, patient samples and in vivo in CRC animal models [25, 30, 32]. Furthermore, as we have shown that iTregs formed upon chronic antigen stimulation express COX-2, secrete PGE2 and suppress effector cells through the cAMP pathway as discussed above [30], this nicely links in vivo immune regulation to molecular mechanisms and points to targeting COX with available drugs as an attractive possibility.

To examine the role of iTreg- and PGE2-mediated suppression of anti-tumour immunity in CRC, we first conducted an observational study on patients referred for surgery of primary CRC. Looking at tumour specimens and peripheral blood samples from patients with CRC, we found significantly elevated PGE2 levels and suppressed anti-tumour immune responses that could be reversed by removal of CD25+ iTreg or by pharmacological perturbation of the COX-2-PGE2-cAMP pathway at the level of COX or PKA [25]. This was the first demonstration of Treg-mediated suppression of anti-tumour immunity in CRC and indication that pharmacological intervention with NSAIDs could block that effect.

About 50% of patients with CRC develop metastatic disease and this population has a worse prognosis than the remaining patients that do not display recurrence following surgical removal of the primary tumour. The liver is the primary site for CRC metastasis and about 30–40% of the patients are available for surgery with primary liver resection or subsequent to down-sizing with neoadjuvant chemotherapy, which has significantly improved the life expectancy in this group of patients. However, the population of resected patients is heterogeneous and while some are cured, others show a rapid recurrence and progression of disease despite a resection with curative intent [3]. Statistically, within 5 years, approximately 60–80% of the liver resected patients will suffer from recurrent metastatic disease, and the majority of the recurrences will occur within the first 2 years after treatment. It was therefore of interest to examine whether Treg suppression of immune responses had impact on clinical fate. We examined Treg-mediated suppression in CRC patients with liver metastasis undergoing liver resection surgery at Oslo University Hospital. We found that the level of Treg-mediated suppression of anti-CEA tumour immune responses (TNFα, IFNγ) through the PGE2-cAMP pathway at the time of surgery predicts future outcome, as patients with recurrent disease after 18 months had significantly more Treg suppression of anti-tumour responses than patients that did not recur and also presented with elevated PGE2 levels as the disease recurred [26].

Although several studies have shown beneficial effect of ASA on primary prevention of CRC, little has been done to examine the effects of NSAIDS or ASA as secondary prevention after diagnosis of CRC where anti-tumour immune regulation would have more impact, and which could have clinical benefit. However, examination of occurrence of metastatic disease in the meta-analysis of randomized ASA studies revealed lower frequency of metastasis in ASA users, which could account for the reduced mortality [14]. Furthermore, a registry study from the Tayside region in Scotland with 3000 cases looking at post-diagnosis ASA use showed reduced CRC-specific mortality (HR 0.58–0.72, depending on method of analysis and site of cancer), indicating a potentially beneficial effect post-diagnosis as secondary prophylaxis [33]. In total, ten previous studies have assessed the survival benefit of aspirin use after the diagnosis of colorectal cancer. Seven of these studies were included in a meta-analysis conducted by Li et al., which showed a significant overall survival benefit with a HR of 0.84 (95% CI, 0.75 – 0.94) [34]. However, in this meta-analysis, no significant benefit was found for colorectal cancer-specific survival, or for patients using aspirin prior to diagnosis. This was also the case in a meta-analysis by Ye et al., which found an overall survival benefit with an HR of 0.74 (95% CI, 0.62–0.89), but no significant advantage with regard to colorectal cancer-specific survival, with an HR of 0.74 (95% CI, 0.51–1.10) [35]. A review on the role of ASA in gastrointestinal oncology concluded that more evidence is required as to whether starting ASA after diagnosis of cancer is effective [36]. Weaknesses with these previous studies as well as the meta-analyses that summarized the observations include small sample sizes, unreliable assessment of aspirin use, highly selected study populations and recall bias.

In order to look at the effect of ASA on a large population, we performed a register-based analysis (Norwegian Cancer Registry / Norwegian Prescription Database) from 2004 to 2011 of all patients in Norway diagnosed with CRC (*n* = 29495 of which 23,162 met criteria for inclusion in the study) and divided these in ASA (*n* = 6109) and non-ASA (*n* = 19535) users. Our data show a 15% reduced cancer-specific mortality in CRC patients taking ASA (> 6 months) compared to non-ASA users with CRC [37]. Furthermore, patients also taking ASA prior to their CRC diagnosis had even further reduced risk (to HR 0.76).

Studies have described an effect of ASA treatment in CRC at doses of 0.5–5 tablets of 325 mg/week (HR 0.57) and a stronger effect (HR 0.47) at doses of > 6 tablets. A dose of 650 mg ASA/week has been defined as the lower limit [18]. Studies of the primary preventive effect from ASA have demonstrated an effect at doses as low as 75 mg per day if duration of treatment is long (> 5 years) and with effects evident sooner at higher doses [9–11] and other studies suggesting daily doses of 300 mg [14]. Thus, there is evidence for an effective dose between 100 and 300 mg/day, which led us to choose the 160 mg daily regimen.

Based on the in vitro and in vivo animal work and the two clinical observational studies described above as well as the register-based data, we now have the basis for asking the question of how a clinical intervention with an NSAID or ASA to block PGE2 production will affect secondary prevention after primary cancer (not examined earlier despite many studies on primary prevention). In conclusion, these findings strongly support initiation of a placebo-controlled trial that investigates the role of ASA as adjuvant treatment in CRC patients.

### Objectives {7}

#### Primary objective

- To determine whether treatment with 160 mg ASA (Trombyl) once daily for 3 years can improve disease-free survival (DFS) in patients treated with resection for CRCLM, compared with placebo

#### Secondary objectives

- To determine the effect of 160 mg ASA on time to recurrence (TTR) and overall survival (OS) compared to placebo
- To determine the effect of 160 mg ASA on Health-Related Quality of Life (HRQOL) outcome measures. The related endpoints will be the eight RAND 36-Item Health Survey 1.0 (SF-36) dimension scores as well as physical and mental health summary measures, and the EQ-5D index value

#### Exploratory objectives

- To determine whether 160 mg ASA can improve DFS and OS in patients with mutations in PIK3CA and KRAS
- To determine the cost-effectiveness of 160 mg ASA compared to placebo
- Direct medical-care costs, quality-adjusted life years (QUALYs) and life years gained

### Trial design {8}

The ASAC trial is a multicentre, randomized, double-blinded, placebo-controlled, group-sequential trial.

## Methods: participants, interventions and outcomes

### Study setting {9}

The ASAC trial will include recruit patients from all academic centres in Scandinavia operating patients with CRCLM. An updated list of participating sites can be found on trial website: https://asac.no.

The participating sites are as follows: Norway: Oslo University Hospital, Haukeland University Hospital (Bergen), Stavanger University Hospital, St Olavs University Hospital (Trondheim), University Hospital of Northern Norway (Tromsø); Sweden: Karolinska University Hospital (Stockholm), Sahlgrenska University Hospital (Gothenburg), Linköping University Hospital, Lund University Hospital, Uppsala University Hospital, University Hospital of Umeå; Denmark: Rigshospitalet (Copenhagen), Aarhus University Hospital, Odense University Hospital.

### Eligibility criteria {10}

#### Inclusion criteria

All subjects undergoing liver resection or ablation (radiofrequency or microwave) for CRCLM as a part of a curative intent may be included in the study if they meet one of the following criteria:

- First time CRCLM (synchronous or metachronous)
- For patients with synchronous CRCLM:
- Primary tumour resected before hepatic metastasis
- Synchronous resection of primary CRC and hepatic metastasis
- Hepatic metastasis resected before primary CRC (“liver-first approach”) and the primary tumour operated within 6 weeks after the liver surgery
- Recurrence of CRCLM (not previously included in this trial)
- Primary tumour radically operated / radiated (rectum)
- No extra-hepatic metastases
- Hepatic metastasis resected with a macroscopic (surgical) free resection margin (R0/R1)
- Ambulatory with a performance status ECOG 0-2
- Age 18 years or above
- Signed informed consent and expected cooperation of the patients for the treatment and follow-up must be obtained and documented according to Good Clinical Practice (ICH GCP), and national/local regulations

#### Exclusion criteria

Patients will be excluded from the study if they meet any of the following criteria:

- For patients operated with “liver-first” approach if the primary is not removed within 6 weeks after the liver surgery
- Concomitant use of ASA or other anticoagulants or platelet inhibitors such as warfarin or clopidogrel
- On-going regular use of systemic corticosteroids or NSAIDs
- Inherited or acquired coagulopathy (haemophilia)
- Blood platelets (thrombocytes) < 100 × 10^9^/L
- Severe heart failure (classified as NYHA class > III)
- Severe kidney failure > grade III
- Liver cirrhosis with a Child-Pugh score > B7
- Known alcoholism
- Pregnancy or breastfeeding
- Previous malignancies within past 5 years, except non-melanomatous skin or non-invasive cervical cancers
- Contraindication listed on the Summary of Product Characteristics (SmPC) of Trombyl:
- Hypersensitivity/allergies to ASA
- Thrombocytopenia
- Previous severe gastrointestinal haemorrhage/peptic ulcer due to ASA/NSAIDs
- Active peptic ulcer
- Haemophilia
- Liver cirrhosis
- Severe congestive heart failure
- Need to use concomitant medications contraindicated according to SmPC of Trombyl

### Who will take informed consent? {26a}

Informed consent must have been given voluntarily by each subject and signed by the patient and an investigator, before any study-specific procedures are initiated. Study nurse or treating surgeon will be obtaining the informed consent.

### Additional consent provisions for collection and use of participant data and biological specimens {26b}

The patients will also sign informed consent for use of clinical and biological data in ancillary studies.

## Interventions

### Explanation for the choice of comparators {6b}

The ASAC study is a placebo-controlled trial comparing Trombyl (ASA) 160 mg o.d. with placebo.

### Intervention description {11a}

Trombyl tablets, 160 mg, or placebo will be administered per orally in one daily dose. The treatment will be continued for a maximum of 36 months or until verification of disease recurrence (primary endpoint), measured by CT or other relevant radiography.

### Criteria for discontinuing or modifying allocated interventions {11b}

Patients may be discontinued from the study at any time if, in the opinion of the investigator, it is medically necessary, or if it is the expressed wish of the patient. Patients are free to discontinue their participation in the trial at any time. Discontinued patients must also discontinue treatment. Specific reasons for discontinuing a patient for this study are as follows:

- Voluntary discontinuation by the patient who is at any time free to discontinue his/her participation in the study, without prejudice to further treatment
- If further study participation (even with treatment discontinuation) is regarded as a liability to the patient with respect to safety and well-being
- Incorrect enrolment i.e., the patient does not meet the required inclusion/exclusion criteria for the study, prior to randomization. Incorrectly enrolled randomised patients should be kept in the study to comply with the intention to treat principle
- Patient lost to follow-up

### Strategies to improve adherence to interventions {11c}

These are not applicable.

### Relevant concomitant care permitted or prohibited during the trial {11d}

All participants enrolled in the trial are not permitted to start using NSAIDs or ASA. In case of medical condition indicating treatment with anticoagulants or platelet inhibitors, the interventional drug will be discontinued.

### Provisions for post-trial care {30}

These are not applicable.

### Outcomes {12}

The primary outcome of the ASAC trial is disease-free survival (DFS) in patients treated for CRCLM. This will be determined by CT scans every 6 months after treatment for CRCLM. The secondary outcomes are time to recurrence and overall survival, and the effect of 160 mg ASA on Health-Related Quality of Life outcome measures. The related endpoints will be the eight RAND 36-Item Health Survey 1.0 (SF-36) dimension scores as well as physical and mental health summary measures, and the EQ-5D index value.

### Participant timeline {13}

**Fig. 1:**
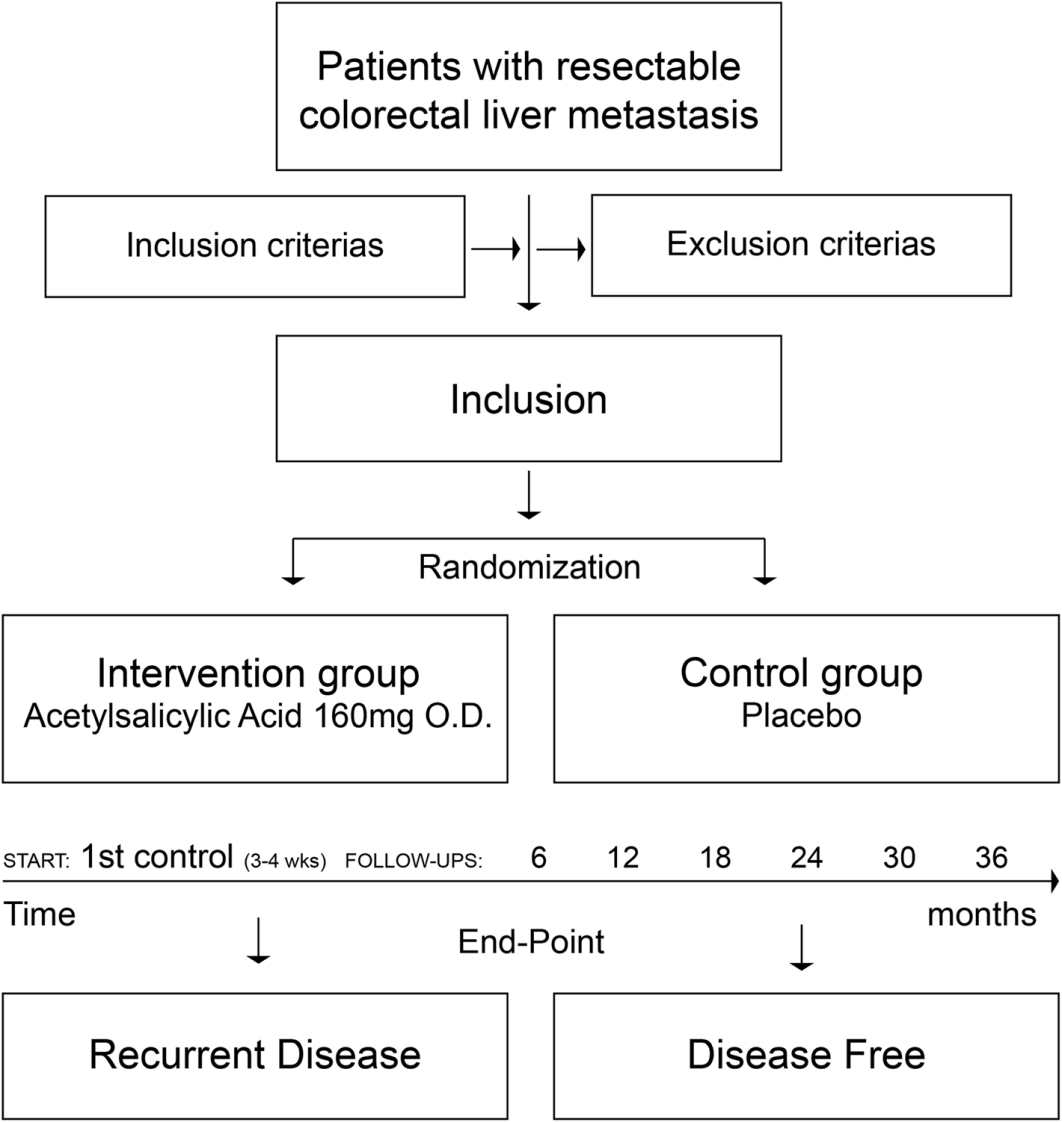
Flowchart of the ASAC trial

**Fig. 2:**
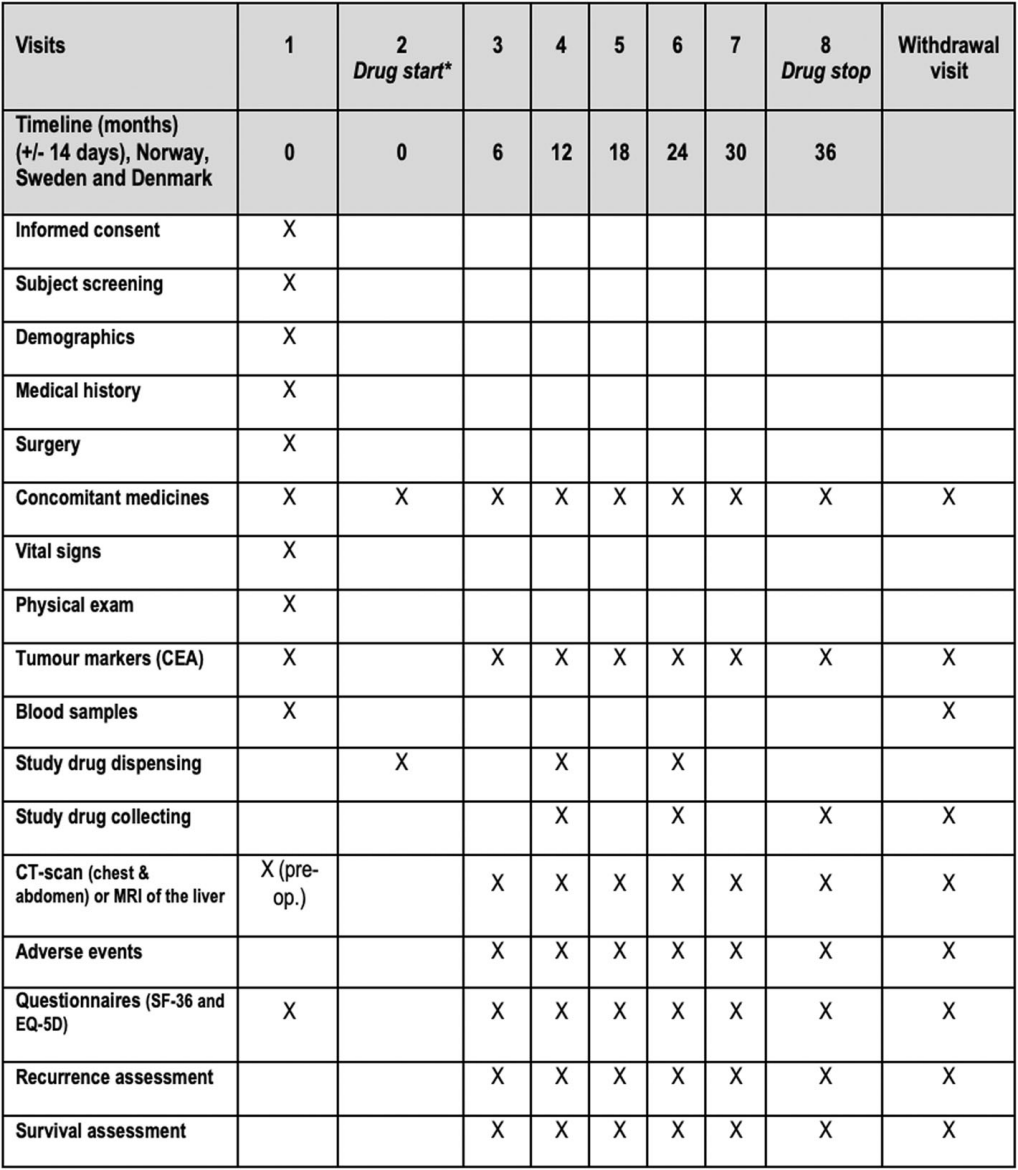
Schematic overview of timeline for patients included in the ASAC trial

The recruitment time of the trial will be 6 years and all included patients will receive active drug or placebo for 3 years. Figure 1 shows a flowchart of the trial and Fig. 2 shows a schedule for patient enrolment, intervention and assessment.

### Sample size {14}

The trial will include 800 patients; 400 in ASA group and 400 in placebo group.

The sample size estimation for this study is based on the following assumptions: two-sided test with a 5% significance level, power: 80%, treatment allocation ratio: 1:1, follow-up time: 36 months, one interim efficacy analysis with an O' Brien-Flemming-like alpha-spending function, disease recurrence rate in the placebo group after 36 months: 40%. Assuming a hazard ratio of 0.7, a total of 254 events (disease recurrences) are required to be 80% sure to reach a statistically significant difference between the treatment groups at the 5% level using a fixed sample size calculation. Adjusting for an inflation rate of 1.06 due to the planned interim analysis, we need 270 events during the study if the study is not stopped at the interim analysis for efficacy. With an approximate overall recurrence rate of 35% and adjusting for loss to follow-up, a total of 800 patients (400 in each arm) will be randomised.

### Recruitment {15}

The trial will recruit eligible patients in Norway, Sweden and Denmark, targeting a population of above 20 million persons. All tertiary centres in Scandinavia will screen patients referred with CRCLM for eligibility to the trial. Study nurses and PI will have regular briefing of recruiting sites to ensure enrolment of patients.

## Assignment of interventions: allocation

### Sequence generation {16a}

Eligible patients will be allocated in a 1:1 ratio between ASA 160 mg and placebo, using a computer randomization procedure stratified by centre. The randomization will be blocked within each stratum. Details of block size and allocation sequence generation will be provided in a separate document that is unavailable to those who enrol patients or assign treatment. The time of randomization will be slightly different for patients with “liver-first” approach. Patients who are already operated for primary CRC or are synchronously operated for primary and hepatic metastases will be randomized postoperatively after the liver surgery. The “liver first” patients will be randomized after the surgery for the primary CRC.

### Concealment mechanism {16b}

Each centre will be provided with batches of treatment kits on a regular basis. Each container will be identified by a kit number and contain 100 tablets. Four containers are sufficient for 1-year use and will have the same kit number. When a patient is deemed eligible and ready for randomization, the investigator will receive the patient kit number through the eCRF system upon randomization. Four containers with the corresponding kit number are then given to the patient. During the study, patients will be given new containers with the same kit number, but with adequate expiry date. Details of given kit numbers are recorded in the eCRF.

### Implementation {16c}

The allocation sequences will be computer generated and eligible patients will be enrolled by the treating liver surgeon or a study nurse who will assign the patients to proper intervention according the abovementioned computer-generated allocation.

## Assignment of interventions: blinding

### Who will be blinded {17a}

The ASAC trial is a double-blinded placebo-controlled trial and following the randomization, the patient, investigator, and clinical physician will be blinded.

### Procedure for unblinding if needed {17b}

In case of a rare emergency where, in the opinion of the investigator, discontinuation of the study treatment is not sufficient and the study treatment must be unblinded in order to evaluate further course of action, the investigator should contact the principal investigator in Norway who will be available 24/7 during the trial period.

## Data collection and management

### Plans for assessment and collection of outcomes {18a}

All included patients will be monitored with a CT scan of the chest and abdomen every 6 months till end of study period (3 years). This is in line with the National guidelines for follow-up of patients operated for CRCLM. New metastases or disease recurrence found on CT scans will be registered in the eCRF and the patient will be discussed on MDT liver meeting for further treatment. The date of disease recurrence will be registered, which is the date of CT/MR scan taken. The assessment of disease recurrence will be done until study end (36 months) or until withdrawal of consent. The observation will be censored at this time point. Disease-free survival is defined as the time from randomization until disease recurrence/new metastases or death from any cause. Safety will be monitored by collection of adverse events at every visit. Demographic and baseline characteristics will be recorded for all included patients. All relevant medical oncological history will be recorded in the eCRF. Molecular profiling of the tumours (both primary and metastases) to assess for gene alterations like mutations in PIK3CA will be recorded as well as immune profile of the tumours, if available. The study subjects will complete a short-form health survey SF-36 and EQ-5D on every study visit according to Fig. 2.

### Plans to promote participant retention and complete follow-up {18b}

Patients who withdraw or are withdrawn from the study will stop further treatment. However, the patients will be followed up according to the National Guidelines for controls following treatment of CRCLM.

If possible, a final assessment will be made (end of study visit). The reason for discontinuation shall be recorded.

Patients who withdraw from treatment should follow all study procedures except those regarding treatment, CT/MR scan, CEA, safety reporting. The patients will be encouraged to continue to complete the SF-36/EQ-5D-5L questionnaires. The reason for study treatment discontinuation will be recorded.

### Data management {19}

The designated site staff will enter the data required by the protocol into the e-case report forms (eCRF). The principal investigator at each participating site is responsible for assuring that data entered into the eCRF is complete, accurate, and that entry is performed in a timely manner. The signature of the investigator will attest the accuracy of the data on each eCRF. If any assessments are omitted, the reason for such omissions will be noted on the eCRFs. Corrections, with the reason for the corrections, will also be recorded/tracked in the eCRF.

The Clinical Data Management System (CDMS) used for the eCRF in this study is Viedoc^TM^. The setup of the study-specific eCRF in the CDMS will be performed by department of clinical research support, Oslo University Hospital.

After database lock, the investigator will receive a digital copy of the subject data for archiving at the investigational site.

### Confidentiality {27}

The investigator will arrange for the secure retention of the patient identification and the code list. Patient files shall be kept for the maximum period of time permitted by each hospital. The study documentation (eCRFs, Site File, etc.) shall be retained and stored during the study and for 15 years after study closure. All information concerning the study will be stored in a safe place inaccessible to unauthorized personnel.

Data management will be performed by the department of clinical research support, Oslo University Hospital. The Data management procedures will be performed in accordance with the department’s SOPs and ICH guidelines. After database closure, the data will be stored in a dedicated and secured area at OUH. Data will be stored in a de-identified manner, where each study participant is recognizable by his/her unique trial subject number. The data will be archived for the time period requested by the competent authorities.

### Plans for collection, laboratory evaluation and storage of biological specimens for genetic or molecular analysis in this trial/future use {33}

In all patients operated for CRC liver metastases, the specimen with tumour is routinely fixated in formalin at the local pathology department. The formalin-fixated tumour is sectioned and stained, and the tumour content in the selected piece is estimated. From patients included in the ASAC trial, there will be selected six [6] slices á 10 μm of tumour for genetic analysis.

In addition to a panel of gene analysis of the tumour, mutation analysis of the BRAF, KRAS and NRAS genes and Mismatch Repair (MMR) status (Microsatellite instability, MSI, Immunohistochemistry, IHC, for MMR genes) will be performed on the selected tumour sections. All samples will be stored for 15 years before they will be destructed.

Blood samples will be taken at time of inclusion and at withdrawal visit to determine haemoglobin, leucocytes, thrombocytes, creatinine, ASAT, ALAT, bilirubin, CRP and CEA. At other visits, the local laboratories will analyse blood samples for CEA. A blood sample will be taken at time of surgery for extraction of germline DNA to be used for the molecular profiling.

## Statistical methods

### Statistical methods for primary and secondary outcomes {20a}

The primary efficacy endpoint, time from randomization to disease recurrence or death by any cause and all time-to-event secondary endpoints will be analysed using a stratified log-rank test accounting for the stratification factor (study centre), and the treatment effect will be estimated using the Cox proportional hazards regression model stratified by study centre. Type of patient (“liver first” or “primary CRC first / synchronously primary CRC and CRCLM”) will be adjusted for in the analysis.

As exploratory analyses, these endpoints will also be analysed using stratified Cox proportional hazards models adjusted for other baseline covariates considered to be of potential prognostic value such as clinical classification, type of chemotherapy, number and size of metastases and tumour biomarker analysis.

A subgroup analysis will be performed to assess the interaction effect between ASA and the mutations in PIK3CA and KRAS. The analysis will be performed on primary and secondary time-to-event endpoints using a stratified Cox proportional model with an ASA/mutation in PIK3CA/KRAS mutation interaction term.

All time-to-event endpoints will be summarized using Kaplan-Meier/Nelson-Aalen plots, estimates of median, 25th and 75th percentiles and hazard ratios.

### Interim analyses {21b}

An interim analysis will be performed when approximately half of the planned primary events (135) have occurred and the primary endpoint has been entered, verified and validated according to the data management plan. A separate document (the Data Monitoring Committee (DMC) charter) details the procedures for the interim analysis. A report will be written following the interim analysis, describing any deviations from the planned analysis, and a recommendation to either continue or stop the study for efficacy. No efficacy information such as hazard ratios, confidence intervals or *p* values will be presented in the report. If the DMC recommends stopping the study for efficacy, the sites will be given notice and no more patients will be randomized. All remaining data must be entered, verified and validated according to the data management plan. The database will then be locked for further entering or altering of data. The allocation list will be opened, and final analyses will be performed. Patients will be informed of their allocation, and patients having received placebo will be offered ASA treatment. If the DMC recommends continuing the study, the final analysis will be performed when the planned primary events (270) have occurred and all data have been entered, verified and validated according to the data management plan.

### Methods for additional analyses (e.g. subgroup analyses) {20b}

The cost-effectiveness of 160 mg ASA compared to placebo will be determined as well as direct medical-care costs, quality-adjusted life years (QUALYs) and life years gained. The eight RAND 36-Item Health Survey 1.0 (SF-36) and the EQ-5D index value will be used to assess QUALYs.

### Methods in analysis to handle protocol non-adherence and any statistical methods to handle missing data {20c}

There will be no handling of missing data for the time-to-event endpoints. Patients without event will be censored at the last point of contact.

### Plans to give access to the full protocol, participant level-data and statistical code {31c}

The protocol is available for the public, but dataset and statistical codes will not be made publicly available to protect confidentiality of the participants.

## Oversight and monitoring

### Composition of the coordinating centre and trial steering committee {5d}

Not applicable.

### Composition of the data monitoring committee, its role and reporting structure {21a}

A data monitoring committee (DMC) is appointed consisting of clinicians and a biostatistician that are independent of the trial. The DMC will perform the interim analysis where they will analyse safety of the trial as well as efficacy of ASA compared to placebo. A clinical study monitor will visit all investigating sites on a regular basis. The monitor will review the relevant CRFs for accuracy and completeness and will ask the site staff to adjust any discrepancies as required. When the responsible study monitor has checked and verified the CRFs, the data will be managed at the Oslo University Hospital scientific server for further handling and statistical evaluation. The monitor is independent from the sponsor.

### Adverse event reporting and harms {22}

All adverse events (AE) and serious adverse events (SAE) that should be reported will be recorded in the patient’s CRF. SAEs must be reported by the investigator to the sponsor within 24 h after the site has gained knowledge of the SAE. Every SAE must be documented by the investigator on the SAE pages (to be found in the CRF). The Serious Adverse Event Report Form will be reviewed by the sponsor designee, the Head of Surgical Clinic, OUH who will evaluate expectedness and relation to IMP. In case of suspected unexpected serious adverse reaction (SUSARs), the report will be sent to Clinical Trial Unit at OUH for further reporting to competent authorities, if applicable. The initial report shall promptly be followed by detailed, written follow-up reports if necessary. The initial and follow-up reports shall identify the trial subjects by unique code numbers assigned to the latter. The sponsor keeps detailed records of all SAEs reported by the investigators and performs an evaluation with respect to seriousness, causality and expectedness. The sponsor will inform all participating sites and principal investigators about any occurred SAEs related to the investigational product.

### Frequency and plans for auditing trial conduct {23}

Each site will be monitored by a study monitor twice a year unless less than 10 patients are recruited since the last monitoring visit. Each site will be monitored at least once per year. Authorized representatives of a Competent Authority and Ethics Committee may visit the centre to perform inspections, including source data verification. Likewise, representatives from the sponsor may visit the centre to perform an audit. The purpose of an audit or inspection is to systematically and independently examine all study-related activities and documents to determine whether these activities were conducted, and data were recorded, analysed and accurately reported according to the protocol, Good Clinical Practice (ICH GCP) and any applicable regulatory requirements. The principal investigator will ensure that the inspectors and auditors will be provided with access to source data/documents.

### Plans for communicating important protocol amendments to relevant parties (e.g. trial participants, ethical committees) {25}

All protocol modifications will be communicated to relevant parties, Competent Authority and Ethics Committee and all trial participants.

### Dissemination plans {31a}

Upon study completion and finalization of the study report, the results of this study will either be submitted for publication and/or posted in a publicly assessable database of clinical study results. The results of this study will also be submitted to the Competent Authority and the Ethics Committee according to EU and national regulations. All personnel who have contributed significantly with the planning and performance of the study (Vancouver convention 1988) may be included in the list of authors.

## Discussion

### Trial status

The trial started recruitment December 2017 and has included 360 patients (October 2020). The recruitment is planned to be completed June 2023 and completion of follow-up in June 2026. Trial Protocol version 5.3. EudraCT number 2014-003601-15. ClinicalTrials.gov Identifier: NCT03326791.

## Data Availability

The ASAC trial team including the trial statistician has full access to the trial data.

## Abbreviations

ASA: Acetylsalicylic acid
APC: Adenomatous polyposis coli
CRC: Colorectal cancer
CRCLM: Colorectal cancer liver metastases
cAMP: Cyclic AMP
COX: Cyclooxygenase
NSAIDs: Non-steroidal anti-inflammatory drugs
PI3K: Phosphatidylinositol 3-kinase
PGE2: Prostaglandin E2
Tregs: Regulatory T cells
